# Hydrogel scaffolds promote neural gene expression and structural reorganization in human astrocyte cultures

**DOI:** 10.7717/peerj.2829

**Published:** 2017-01-11

**Authors:** V. Bleu Knight, Elba E. Serrano

**Affiliations:** Department of Biology, New Mexico State University, Las Cruces, NM, United States; Cell Decision Process Center, Massachusetts Institute of Technology, Cambridge, MA, United States

**Keywords:** 3D culture, RNA-seq, Tissue engineering, Transcriptome, Hydrogel, Astrocyte, Gene expression

## Abstract

Biomaterial scaffolds have the potential to enhance neuronal development and regeneration. Understanding the genetic responses of astrocytes and neurons to biomaterials could facilitate the development of synthetic environments that enable the specification of neural tissue organization with engineered scaffolds. In this study, we used high throughput transcriptomic and imaging methods to determine the impact of a hydrogel, PuraMatrix™, on human glial cells *in vitro*. Parallel studies were undertaken with cells grown in a monolayer environment on tissue culture polystyrene. When the Normal Human Astrocyte (NHA) cell line is grown in a hydrogel matrix environment, the glial cells adopt a structural organization that resembles that of neuronal-glial cocultures, where neurons form clusters that are distinct from the surrounding glia. Statistical analysis of next generation RNA sequencing data uncovered a set of genes that are differentially expressed in the monolayer and matrix hydrogel environments. Functional analysis demonstrated that hydrogel-upregulated genes can be grouped into three broad categories: neuronal differentiation and/or neural plasticity, response to neural insult, and sensory perception. Our results demonstrate that hydrogel biomaterials have the potential to transform human glial cell identity, and may have applications in the repair of damaged brain tissue.

## Introduction

The limited capability of the mammalian nervous system to repair itself after trauma or disease-related degeneration has inspired the design of engineered biomaterials that encourage the growth of neurons and glia in matrix environments that more closely resemble the tridimensional (3D) native tissue. Such biomaterials can support the expansion and differentiation of immature cells into mature neurons and glia that could potentially be used for transplantation into humans in order to improve diseased phenotypes or repair damaged tissue (Prang et al., 2006; Yim, Pang & Leong, 2007; Ashton et al., 2007; Wang et al., 2010a; Ortinau et al., 2010; Hjelm et al., 2013; Bozza et al., 2014; Li et al., 2014). In addition to sculpting tridimensional growth, biomaterials can be engineered to modulate cellular mechanical and chemical properties that specifically promote neural cell survival (Katz & Burdick, 2009; Wang et al., 2010b; Jeffery et al., 2014). For example, studies have illustrated that the presence of biodegradable matrices at the site of injury can ameliorate reactive astrogliosis and glial scarring, processes that pose a significant barrier to central nervous system (CNS) recovery after trauma (Tysseling-mattiace et al., 2008; Ansorena et al., 2013; Placone et al., 2015). The capacity of the 3D microenvironment to impact cellular morphology as well as transcript and protein expression has been demonstrated for neurons and glia of primate and rodent origin (Georges et al., 2006; Teixeira et al., 2009; Puschmann et al., 2013; Sur et al., 2013; Levy et al., 2014). However, the potential differences between human and rodent astrocytes warrant further investigations with astrocytes of human origin (Oberheim et al., 2009; Levy et al., 2014).

In this study we investigated the consequences of growth in a self-assembling peptide hydrogel environment on human astrocytes. We selected the normal human astrocyte cell line (NHA; Lonza) for this research because it is a primary derived cell line of early developmental origin that retains the ability to differentiate into neuronal cells (Azizi & Krynska, 2013). We assessed how a hydrogel environment affects culture morphology and global gene expression with the intention of determining how biomaterials can potentially affect neural and neuroglial cell fate in humans. We implemented RNA sequencing (RNA-seq) technology for genetic analysis because high throughput transcriptomic approaches are rapidly advancing our ability to identify cell types and subtypes based on classic “marker gene” expression as well as through the identification of novel genes that are expressed in distinct cell types (Cahoy et al., 2008; Puschmann et al., 2013). The well annotated human genome and a rich body of prior literature allowed us to mine the RNA-seq data for functional significance (Ogata et al., 1999; Apweiler et al., 2004; Smith et al., 2007; Huang, Sherman & Lempicki, 2009a; Safran et al., 2010; Szklarczyk et al., 2014; Gray et al., 2015).

A matrix environment was constructed using the hydrogel, PuraMatrix™, which has an elasticity coefficient similar to brain tissue (Spencer, Cotanche & Klapperich, 2008). For comparison, NHA were cultured in a conventional monolayer environment on tissue culture polystyrene (TCPS). Live cultures were imaged with phase contrast optics prior to RNA extraction for RNA-seq analysis. Using open-source bioinformatics tools and manual curation, we completed an ontological characterization of highly expressed genes, expressed genes with established function in the nervous system, as well as genes that were differentially expressed between the two culture conditions. Immunolabelling and confocal imaging experiments were used to evaluate the distribution of class III *β*-tubulin in TCPS- and hydrogel-cultured cells. Our results show the differences in the structural organization of NHA cells in monolayer and matrix environments and highlight human gene categories and networks that are enhanced through culture in a hydrogel environment. These findings add to the growing body of literature that illustrates the potential of engineered biomaterials to dictate cell fate. Moreover, we provide evidence that human astrocytes can be coaxed into assuming neuronal characteristics through culture with a hydrogel scaffold. The use of commercially available cell lines and biomaterials in our experimental design facilitates the reproduction of our experiments by any laboratory with access to a biosafety level two tissue culture facility, and is intended to accelerate subsequent research that leverages our results.

## Methods

### Cell culture

The Normal Human Astrocyte (NHA; Lonza, CC-2565) cell line was implemented in this study because its neuroglial lineage and species origin present a practical *in vitro* system for studying the effects of hydrogel biomaterials on the human brain. Furthermore, because NHA are a commercially available cell line, they can be used in follow-up and confirmatory experiments by our research team as well as those of other investigators. NHA from two different biological donors (lots #000080982, #000022529; referred to hereafter as Donor A and Donor B) were cultured according to the manufacturer’s specifications. All cytokines, growth factors, and supplements from the SingleQuots™ kit (Lonza; CC-4123) were added to Astrocyte Basal Medium (Lonza; CC-3187). We omitted the manufacturer-recommended gentamicin from cell media because the aseptic techniques used in our laboratory enable NHA culture in an antibiotic-free environment. Frozen ampules of cells were thawed and plated into four T-25 flasks (passage 0) and incubated at 37°C, 5% CO_2_, 275 mOsm. Media were replenished within 24 h of thawing cells, and every 48 h that followed. NHA were subcultured by partial digestion with ReagentPack™ subculturing reagents (Lonza; CC-5034) when cultures reached 80% confluence, five days after plating (passage 1).

### Matrix preparation

We selected a self-assembling 16-mer peptide hydrogel matrix (PuraMatrix™; Ac-RADARADARADARADA-CONH_2_; 3DMatrix Medical Technology, formerly BD Biosciences) for our experiments because it is chemically defined, commercially available, and cells are readily dissociated from the matrix for RNA isolation. The limited biological activity of PuraMatrix™ facilitates the evaluation of effects that occur primarily in response to the microenvironment structure. In particular, experiments have shown that PuraMatrix™ is not immunogenic, cytotoxic, pyrogenic, or hemolytic, and does not bind to cells via integrin receptors (Holmes et al., 2000; Zhang, Ellis-behnke & Zhao, 2005). Moreover, physiological conditions are retained in PuraMatrix™ because the osmolarity of the culture medium is not altered by addition of the hydrogel (matrix = 275 ± 2 mOsm; monolayer = 276 ± 4 mOsm; mean + S.D., *n* = 3).

PuraMatrix™ (1%) was vortexed for 30 s, diluted in sterile water to 0.35%, and vortexed again for 30 s. The matrix was centrifuged at 210 G for 5 min to remove bubbles. A total of 125 µl of peptide hydrogel per cm^2^ was added to each culture vessel prior to the induction of gelation. Self-assembly of the microenvironment was initiated by adding an equivalent volume of cell culture media and equilibrating for one hour. In order to bring the environment to a neutral pH, the media were replenished twice over the following hour and allowed to incubate overnight prior to the addition of cells.

### Experimental design

**Figure 1:**
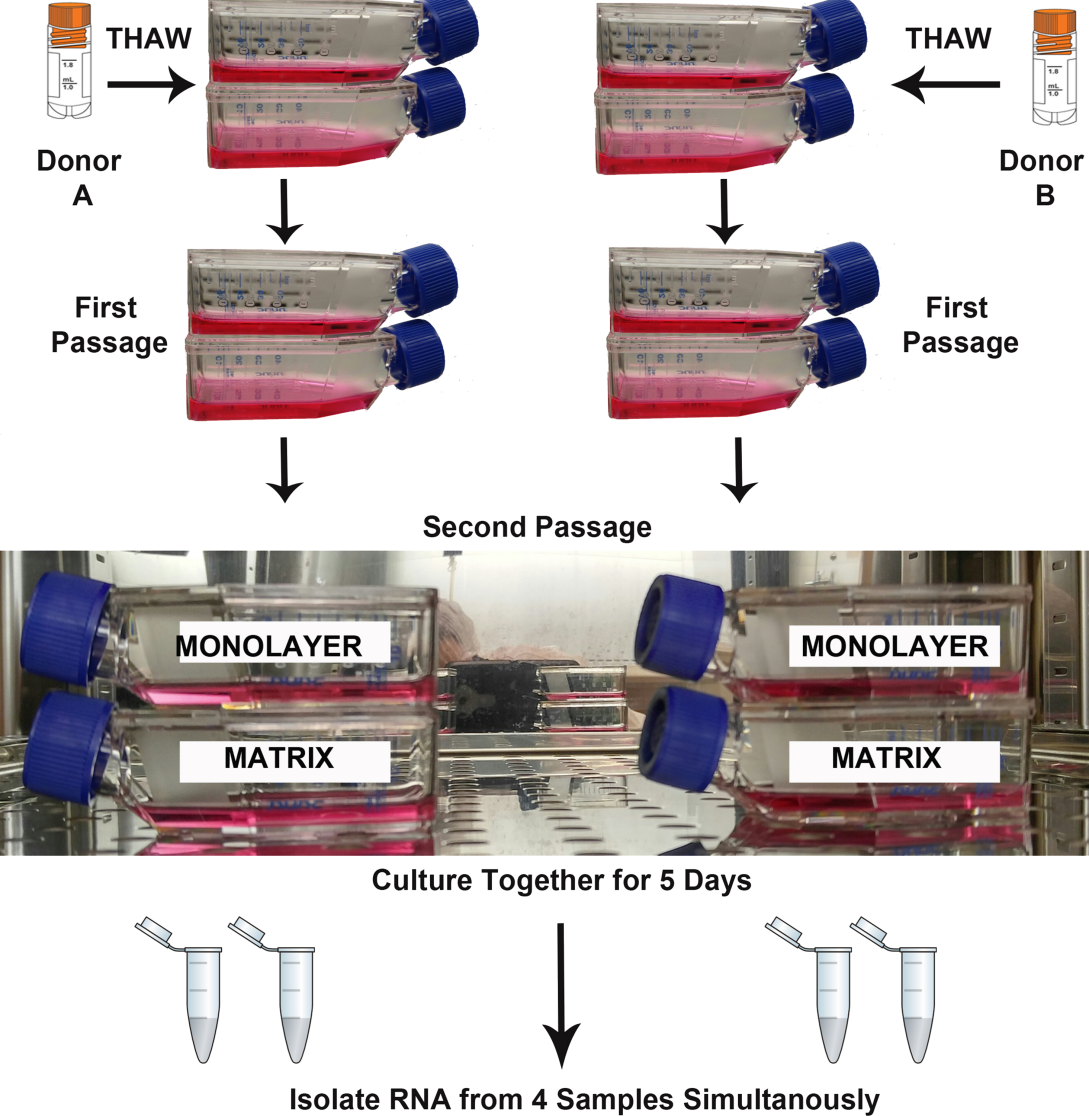
Design of RNA sequencing experiment. The second passages of normal human astrocyte cells from two biological donors were cultured in monolayer and matrix environments for 5 days prior to RNA extraction. Media were replenished on day 3. Cryo vial symbol is courtesy of the symbol library provided by the Integration and Application Network, University of Maryland Center for Environmental Science (ian.umces.edu/symbols/).

Two biological replicates (Donor A, B) were cultured simultaneously under two experimental conditions (TCPS and PuraMatrix™) for RNA isolation and sequencing (Fig. 1). The parallel culture and subsequent RNA isolation from donor cultures maintained in both microenvironments was designed to minimize the false discovery of differences due to technical variation. T-25 flasks for each NHA donor and each experimental condition were seeded with 5,000 cells per cm^2^. As recommended by the vendor, the media were replenished on the 3rd day after plating, and RNA was isolated when monolayer cultures reached approximate confluence (day 5).

### Live cell imaging with phase contrast microscopy

Prior to RNA isolation, phase contrast images of all four live samples were captured with Metavue image capture software (Molecular Devices), which controlled a Coolsnap HQ CCD camera (Photometrics) attached to an inverted Nikon TE-2000 microscope (20X Magnification).

### RNA isolation

RNA isolation was undertaken according to the instructions in the TRIzol^®^ Plus RNA Purification Kit (Ambion). DNA was removed using the DNA-free™ kit (Ambion) according to the instructions provided by the manufacturer. RNA quality was assessed with an Agilent 2100 Bioanalyzer.

### Library preparation and sequencing

RNA samples with RIN Values greater than 8.8 were submitted to the BioMicro Center at the Massachusetts Institute of Technology for library preparation and sequencing (Fig. S1). A 500 ng aliquot of total RNA (500 ng) from each sample was poly-A purified and converted to cDNA using the manufacturer’s instructions for the Illumina TruSeq RNA Sample Preparation Kit. Samples were fragmented on the SPRI-works system using BioMicro Center adapters, barcoded for multiplexing, enriched using BioMicro Center PCR primers, and assessed for fragment size and distribution on an Agilent 2100 Bioanalyzer. Samples were multiplexed and sequenced on an Illumina HiSeq 2000 (Illumina) in accordance with the protocol for 50 base pair (bp) paired end (PE) reads. Phred scores and nucleotide composition were assessed with FastQC to evaluate base call accuracy (Figs. S2 and S3). All four Illumina libraries were multiplexed into one lane (to minimize lane variation) and sequenced on two separate runs of the HiSeq 2000. RNA sequencing (RNA-seq) data collected from all four samples on these two separate occasions are considered technical replicates and labelled as Lane 1 and Lane 2 in the manuscript and data files.

### Statistical and ontological analyses

RNA sequencing data analysis was undertaken with freely available online tools that included open source software and the statistical programming language, R. Illumina reads were aligned to the UCSC human genome (hg19) using TopHat software (Version 2.0.8b) which is made freely available on GenePattern by the Broad Institute (Langmead et al., 2009). The countOverlaps function in the R package Genomic Ranges (version 1.16.4) was used to assemble transcripts and estimate abundance (Lawrence et al., 2013). The abundance estimates produced by Genomic Ranges are not normalized and therefore referred to as raw data throughout this manuscript. Cuffdiff software (Version 2.1.1), made freely available on GenePattern by the Broad Institute, was used to normalize transcript abundance by the effective library size and transcript length. Prior to differential expression analysis, the raw read counts for technical replicates were summed to evaluate only the differences between true biological replicates (*n* = 2). The R package DeSeq (version 1.16) was used to evaluate differential expression because of the package’s ability to identify differentially expressed genes (DEGs) even among samples with low numbers of biological replicates (Anders & Huber, 2010). The first generation ‘DeSeq’ algorithm was chosen instead of the newer ‘DeSeq2’ package because ‘DeSeq’ estimates differential expression using a more conservative algorithm that is less likely to reveal false differentially expressed genes (Love, Huber & Anders, 2014). Coding transcript sequences (CTSs) for which DeSeq reported a Benjamini–Hochberg adjusted *p*-value of ≤0.1 and a log_2_ fold change ≥2 met the criteria for differential gene expression. Cufflinks (version 2.2.1) was used to normalize raw read counts by fragments per kilobase of exon per million reads mapped (FPKM) to compensate for bias introduced by transcript length variance (Trapnell et al., 2012).

Functional significance was imparted to highly and differentially expressed genes using DAVID, the Database for Annotation, Visualization, and Integrated Discovery, with medium stringency settings [29,30]. The Gene Ontology Consortium (GO) terms, Kyoto Encyclopedia of Genes and Genomes (KEGG) pathways, as well as keywords and sequence features from Uniprot were analyzed in detail for genes assigned to clusters with an enrichment score > 1.3 (Langmead et al., 2009; Sur et al., 2012; Lawrence et al., 2013). Protein networks were identified with STRING using the high stringency filter and a maximum of 10 interacting nodes (Szklarczyk et al., 2014). The role of DEGs in nervous system function, if any, was established through manual curation by PubMed query of each DEG using logical “AND” with terms “brain” or “neural,” and by searching the text in GeneCards (Safran et al., 2010).

### Immunocytochemistry

Chambered slides with monolayer- and matrix-cultured NHA were rinsed twice in 1 ml/chamber of phosphate buffered saline (PBS) before fixing in an ice cold mixture of methanol and acetone (1:1) for 15 min (1 ml/chamber). Fixed cells were rinsed twice in ice cold PBS, then samples were incubated for one hour at ambient temperature (∼26°C) with 1 ml/chamber of blocking solution of PBS comprising 10% normal goat serum, 1% bovine serum albumin (BSA), 0.3 M glycine, and 0.1% TWEEN^®^ 20. Primary and secondary antibodies were diluted in PBS containing 1% BSA and 0.1% TWEEN^®^ 20 (PBS-ab). Rabbit anti-TUBB3 antibody (Abcam; catalog # ab52623, lot # GR14955-15) was diluted at 1:300 in PBS-ab and 250 µl of antibody solution was incubated with samples overnight at 4°C in a sealed, humidified chamber. Primary antibody was omitted from negative control samples to assess nonspecific binding of the secondary antibody. Cells were rinsed 3 × 5 min in 1 ml/chamber PBS before incubating with 250 µl of Alexa Fluor^®^ 488-conjugated goat anti-rabbit IgG (Abcam; catalogue # 150081, lot # GR282725-1) diluted at 1:500 in PBS-ab for one hour at ambient temperature (∼26°C) in the dark. Cell nuclei were counterstained with the nucleic acid stain Hoescht 33342 (0.2 µg/ ml) diluted in 18 MΩ water. Cells were rinsed 3 × 5 min in PBS before mounting in glycerol and PBS (1:1). Confocal images were acquired with a 10X/0.3 objective mounted on an inverted LSM 700 microscope (Zeiss). Hoescht 33342 and Alexa Fluor^®^ 488 were excited with laser excitation at *λ* = 405 and *λ* = 488, respectively. Main beam splitter was MBS 405/488/555/639 with the SP 555 filter used to collect emission from both fluorophores. Images were captured with settings which excluded fluorescence from control samples incubated with only secondary antibody. Immunolabelling and image capture were repeated in triplicate on the second and third passages of NHA (lot #0000402839). A blind, qualitative image assessment was undertaken by three individuals asked to combine images into groups, if applicable, and describe the staining patterns.

### Figure preparation

Figures were prepared with Wordle (Feinberg, 2014), Excel (Microsoft, 2010), and the ‘ggplot2’ (Wickham, 2009) and ‘pheatmap’ (Kolde, 2015) data visualization packages for the R statistical programming language. Additional image assembly, labelling, and alignment were completed with Photoshop (Adobe CS6). The ZenLightEdition software package (Zeiss 2009) was used to optimize the relative contribution of the fluorescence signal from the emission channels to the merged confocal image, and to export confocal images as JPEG files.

### Responsible conduct and reproducibility

Experimental procedures were developed in accordance with established guidelines for preclinical research (Landis et al., 2012; The National Institute of Health, 2016). NHA were produced by the vendor (Lonza) in compliance with national ethics standards (document available upon request from vendor). Laboratory culture procedures adhered to vendor specifications; cells were used within the maximum 10 population doublings (three passages). RNA-seq results (see below) further confirmed the human origin of the cell line.

The use of the commercial (human) cell lines was approved by the NMSU Institutional Biosafety Committee, approval # 1401SE2F0103, “Gene Expression in the Nervous System.” The protocol was exempt from review by the Institutional Review Board because the commercial cell lines were developed before 2015 and were de-identified by the vendor, Lonza, who retains a signed record of informed consent from human donors. The NIH Extramural Institutional Certification form for human cell lines created before January 25, 2015 was signed by the institutional offices and the principal investigator (EE Serrano) and submitted to NIH with the GEO data files. In accordance with data sharing policies set forth by the NIH, both the raw and processed data are available for download from GEO using the accession number GSE81995.

Official gene symbols were used in accordance with the standards set forth by the HUGO Gene Nomenclature Committee (Gray et al., 2015). Analysis was undertaken with two biological replicates because the vendor provides primary cell lines from two different human donors. A balanced and blocked design of two biological and two technical replicates was implemented to minimize the impact of confounding variables (Auer & Doerge, 2010). For a complete description of the statistical test that was used to evaluate differential expression, see Anders & Huber (2010). Sequence data were collected with a single blind protocol at the MIT BioMicro center, without prior knowledge of the nature of the biological samples. Researchers at NMSU assigned groups and assessed the outcomes using a single blind analysis.

Rabbit anti-TUBB3 monoclonal antibody (Abcam; catalog # ab52623, lot # GR149555-15, RRID:AB_869991) was used for immunocytochemistry with human astrocytes, as validated by the vendor with western blot analysis and confirmed in several references listed on the vendor’s website.

## Results

### Live cell imaging with phase contrast microscopy

**Figure 2:**
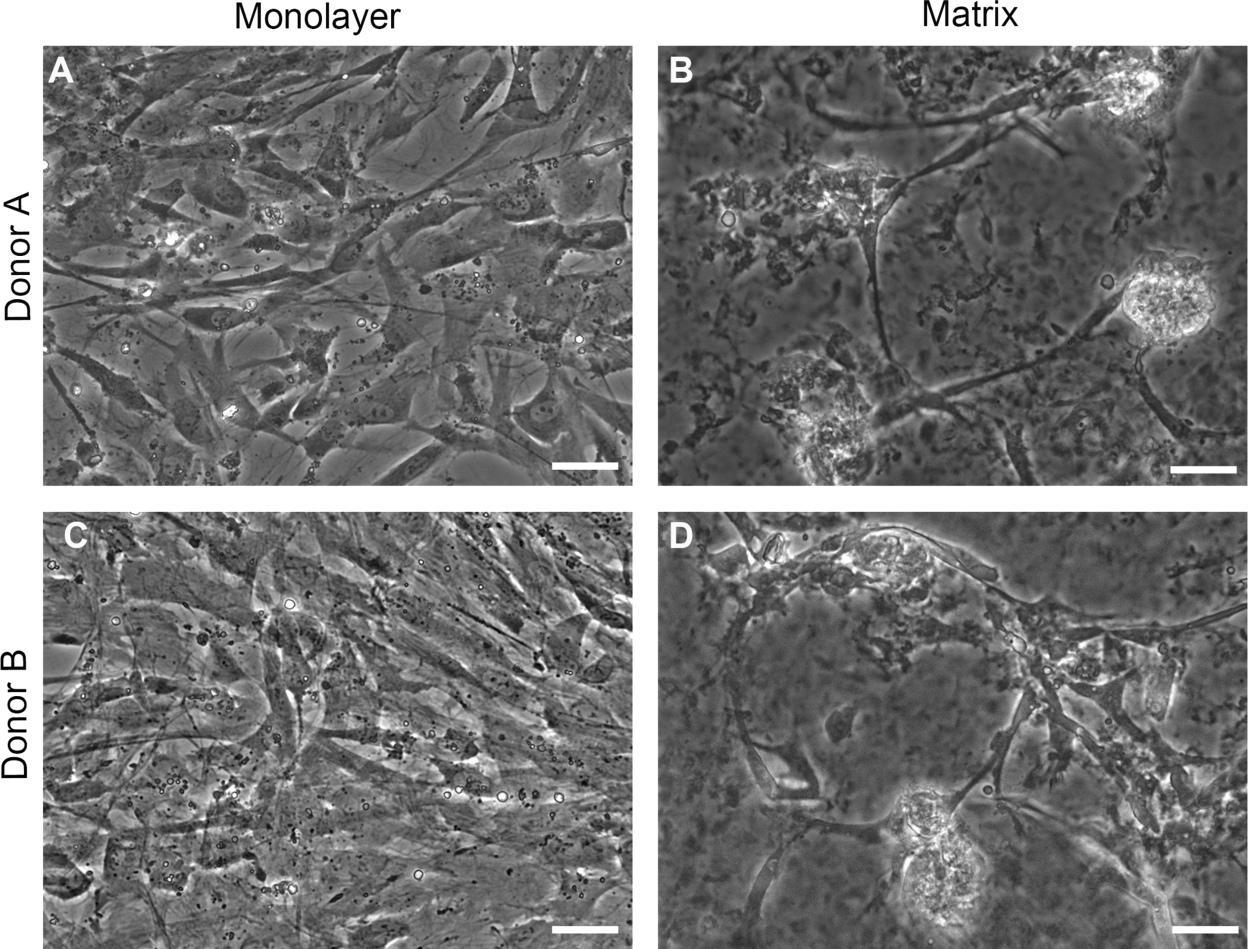
Phase contrast images. Normal human astrocytes cultured in monolayer (A, C) and matrix (B, D) environments for five days and were imaged live, prior to RNA extraction for transcriptome analysis. Representative images are shown for two biological donors: Donor A (A, B); Donor B (C, D). Scale bar = 50 µm.

Phase contrast images of cells on TCPS and with peptide hydrogel demonstrate structural differences between NHA grown in TCPS or hydrogel microenvironments (Fig. 2). NHA cultured on TCPS surfaces (Figs. 2A and 2C) appear uniformly flat and phase dark, as is typical of astrocyte cultures, in comparison to NHA cultured with PuraMatrix™ (Figs. 2B and 2D). In the hydrogel environment, NHA reorganize and form aggregate structures that resemble neuron-glia cocultures. Clusters of phase-bright cell regions that are characteristic of neuronal somata are visible interspersed in the matrix among phase dark astrocytes (Serrano & Schimke, 1990; Abd-el-basset, 2013).

### Evaluation of sequence quality and read alignments

Sequence quality assessment of PE RNA-seq reads from NHA revealed that, for all samples and read positions, the average Phred scores for nucleotides met the criteria for 99% base call accuracy (Phred score > 20; Fig. S2). The percent nucleotide composition values fluctuate during the initial reads (≤13), then converge to a more constant value (25%) as the reads lengthen (Fig. S3). On average, over a hundred million reads reads per replicate sample aligned to the reference human genome (hg19) for a total of 856,790,008 RNA-seq read alignments (Table 1). Approximately 80% of the reads aligned uniquely. Both paired ends aligned to the reference genome (hg19) for 45% of the PE reads, while 2% of alignments were discordant and did not meet the expected distance and/ or orientation constraints established by TopHat.

**Table 1:** RNA-seq alignments to the Human Genome. Paired-end RNA-seq reads from Astrocyte RNA were aligned to the Human Genome (HG19; UCSC) using TopHat (Version 2.0.8b).

Sample	Total reads	Total uniquely aligned reads	% Uniquely aligned	Both ends aligned	Discordant alignments	Pairs with multiple alignments
Monolayer A Lane 1	129052614	102805592	79.7	56807599	1969147	4548539
Monolayer A Lane 2	114220280	83950884	73.5	52070171	3695376	7878121
Matrix A Lane 1	129408746	105438159	81.5	57860404	1864415	4186338
Matrix A Lane 2	113611166	85854999	75.6	52197531	3301865	7133894
Monolayer B Lane 1	100083832	81127497	81.1	44361901	1207636	3280062
Monolayer B Lane 2	92612888	70105954	75.7	42351343	2541256	5705233
Matrix B Lane 1	93772260	76433102	81.5	41399683	1092590	2770120
Matrix B Lane 2	84028222	64455911	76.7	38185638	2164749	4671503
Average	107098751	83771512	78.2	48154284	2229629	5021726

### Normalization and establishment of threshold for expression

**Figure 3:**
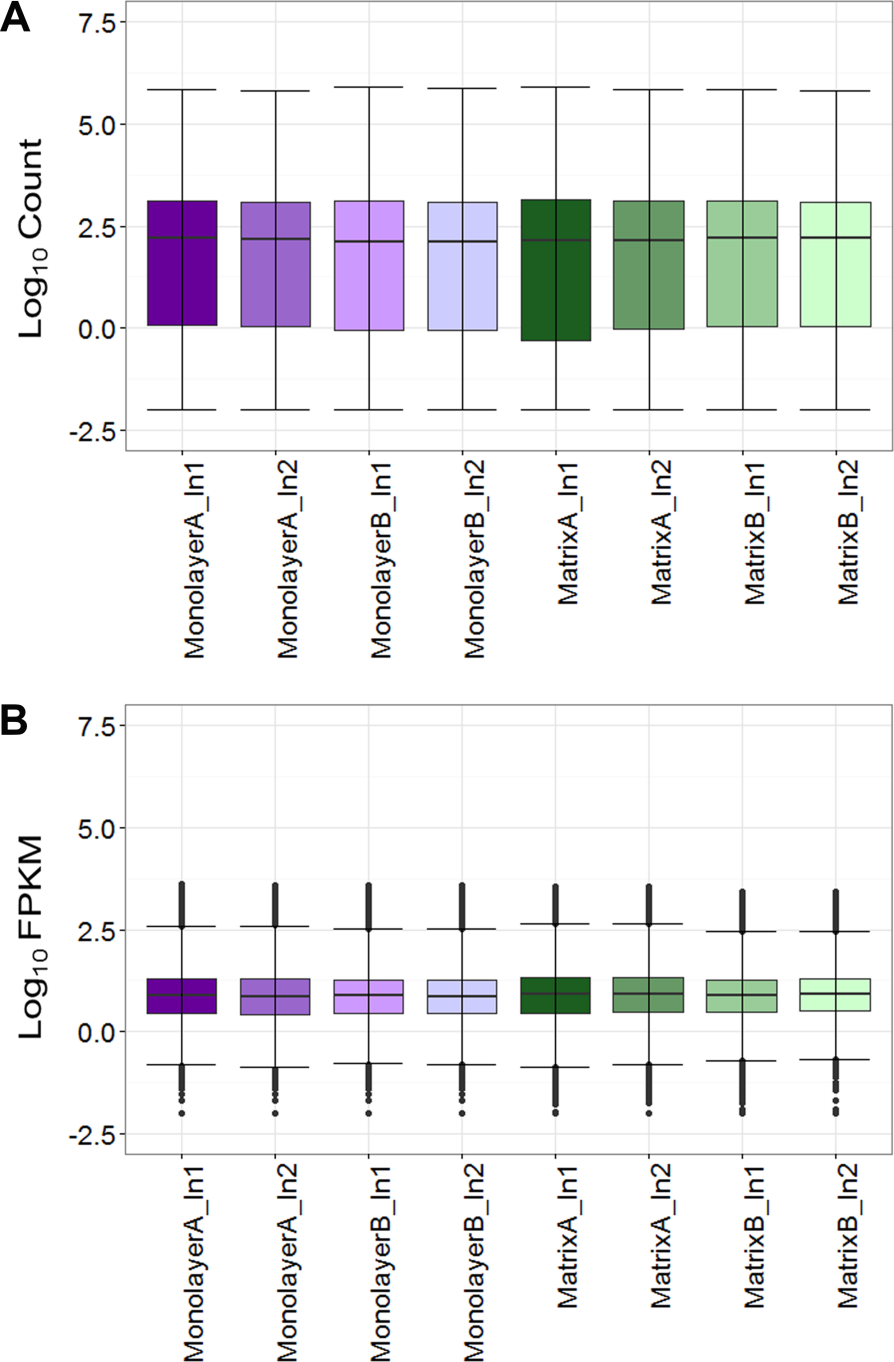
Boxplots of raw RNA-seq alignment counts (A) and thresholded FPKM normalized data (B). Normal human astrocyte RNA samples from two different donors (denoted by A and B) grown in monolayer and matrix conditions were multiplexed for sequencing and replicated on two different lanes (denoted by 1 and 2).

**Figure 4:**
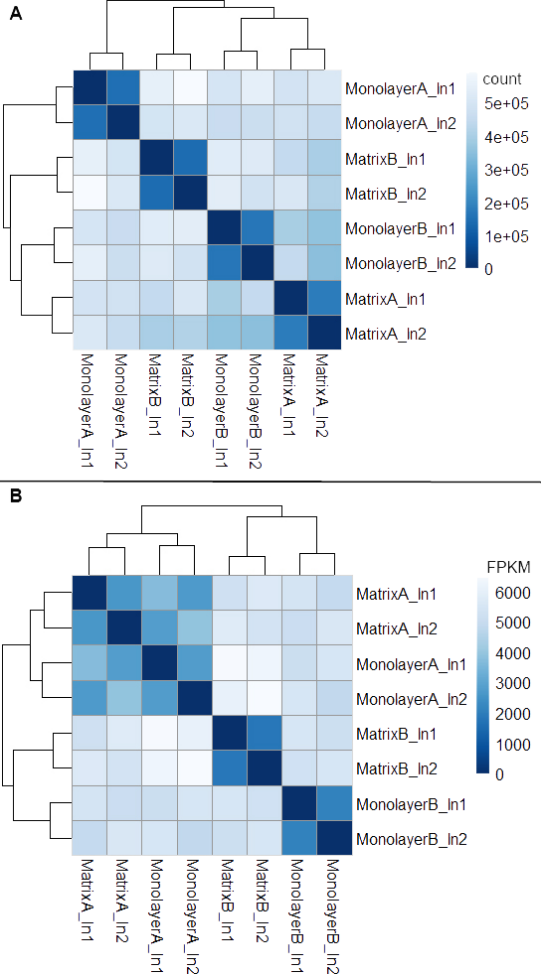
Heat maps of Euclidean sample-to-sample distances and cluster dendrograms of raw RNA-seq alignment count (A) and FPKM normalized data (B). Normal human astrocyte RNA samples from two different donors (denoted by A and B) grown with monolayer and matrix conditions were multiplexed for sequencing and replicated on two different lanes (denoted by ln1 and ln2).

**Figure 5:**
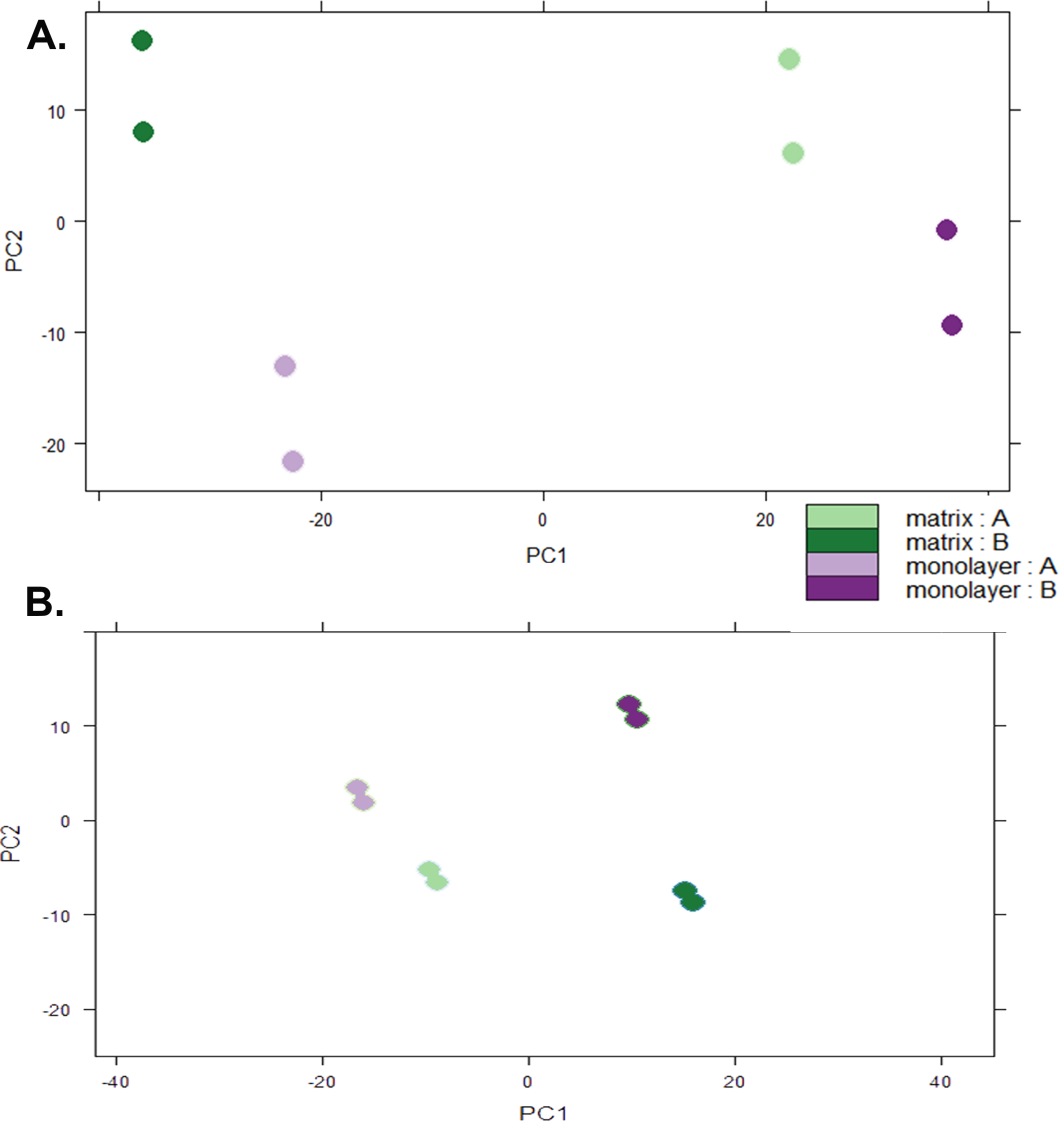
Principal Component Analysis. Plots of raw read count data (A) and FPKM normalized data (B) from RNA sequencing of normal human astrocyte RNA from two different donors (denoted by A and B) grown with TCPS and peptide hydrogel. The pairs of dots correspond to technical replication on two different lanes.

Box plots of log_10_-transformed raw RNA-seq alignment reads and log_10_-transformed FPKM-normalized coding sequences demonstrated consistency between the technical replicates, with slightly more variation between the biological replicates (Fig. 3). The interquartile ranges (25th–75th percentiles) for the raw read data were larger, and count values were greater, as compared to FPKM normalized data. In Fig. 4, the effects of normalization are portrayed in heat maps of Euclidean distances grouped by hierarchical clustering. Inspection of clusters and their internodal distances illustrates that, in both normalized and raw data, the most similar expression patterns are observed among the technical replicates. Cluster analysis of the raw data (Fig. 4A) linked the monolayer sample from donor B closest to the matrix sample from donor A. In contrast, the normalized data (Fig. 4B) illustrate the closer relation of expression between monolayer and matrix samples from the same donor. In Fig. 5, the effects of normalization are reiterated in principal component analyses. Inspection of groups in normalized and raw data revealed that the most similar expression patterns are observed among the technical replicates. Principal component analysis of the raw data (Fig. 5A) grouped the monolayer sample from donor B closest to the matrix sample from donor A. In contrast, the normalized data (Fig. 5B) illustrate the closer relation of monolayer and matrix samples from the same donor.

In order to evaluate genes with estimated mRNA copy numbers greater than one per cell, we set a detectability threshold of FPKM ≥ 1 (Hebenstreit et al., 2011; Nagaraj et al., 2011) and only CTS that met this criterion in at least one sample were eligible for inclusion in our detailed analysis. These criteria excluded 24 CTSs with a status of “NA” (File S1), 2,183 CTSs with all eight FPKM values of 0 or NA (File S2), and 6,447 CTSs with FPKM values from 0.1–1 (File S3). Moreover, 1,806 genes representing noncoding RNA sequences were removed from subsequent analyses (File S4). Application of these filter criteria identified 12,822 CTSs for downstream analyses (File S5).

**Figure 6:**
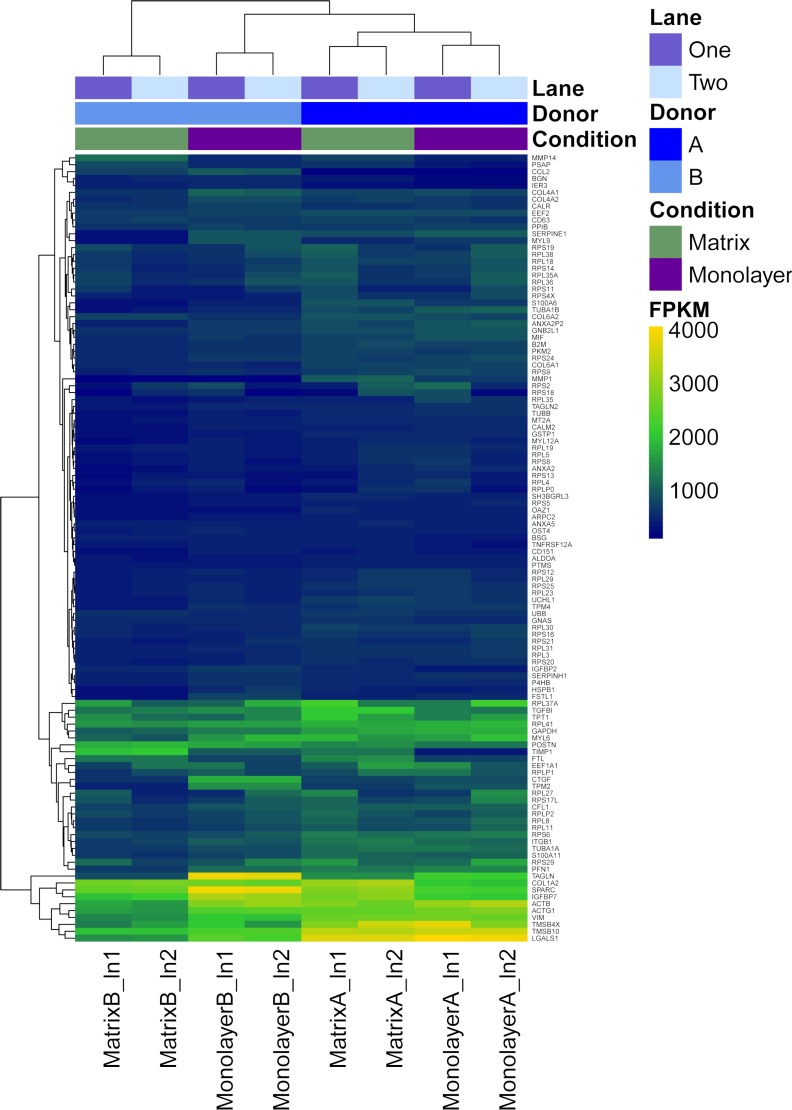
Heatmap with hierarchical clustering for the most expressed genes common to RNASeq datasets from NHA grown in TCPS and in hydrogel environments (*n* = 115). FPKM values for RNA samples from two different donors (A, B) grown with monolayer (purple) and matrix (green) environments, and sequenced on two different lanes (1, 2).

**Figure 7:**
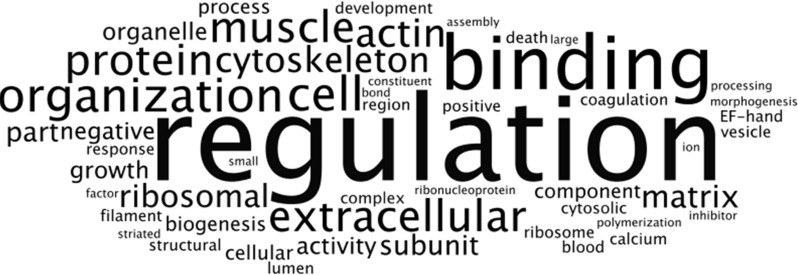
Frequency analysis of DAVID ontological terms for the most expressed genes common to RNASeq datasets from NHA grown in TCPS and in hydrogel environments (*n* = 115). Wordle was used to arrange the DAVID terms that described the nine significant clusters (enrichment scores > 1.3; File S6) of the most highly transcribed CTSs into an image that represents increasing frequency with larger font size.

### Characterization of the most abundant transcripts

First, FPKM values of coding transcript sequences for cells grown in monolayer and matrix conditions were ranked from highest to lowest. Next, we identified the top 10% (*n* = 128) of the 12,822 CTSs with the highest average FPKM for cells grown in monolayer and matrix conditions. Approximately 90% (*n* = 115) of the most highly transcribed CTSs were common to cells grown in monolayer and matrix conditions, while 26 were not common to both conditions. The most highly transcribed common CTSs (*n* = 115) were used to construct a heat map, which depicts the FPKM for the CTSs (Fig. 6). DAVID analysis clustered the most expressed genes common to NHA grown in TCPS and hydrogel environments (*n* = 115) into 22 significant a clusters (enrichment scores > 1.3; File S6). The GO terms corresponding to the most highly transcribed CTSs were arranged into a Wordle image that represents higher frequency with larger font size (Fig. 7; Feinberg, 2014).

A total of 24 of the 26 CTSs that were not common to both conditions were found in the top 15% of the most transcribed genes in the other condition. Two genes, *ACTA2* and *HTRA1*, ranked much lower in the other condition. When CTSs were ranked by highest average FPKM, the CTS for *ACTA2* was ranked #61 for cells grown on TCPS; this ranking fell to #476 (37%) of the highest average FPKM for cells grown in PuraMatrix™. The *ACTA2* gene corresponds to the alpha actin 2 protein, which is involved in the formation of tight junctions in the blood brain barrier (Safran et al., 2010). *ACTA2* was not considered to be differentially expressed between the two conditions using our DEG criteria; however, the log_2_FC value was −2.5. For cells grown in PuraMatrix™, the CTS for *HTRA1* was ranked #122 of the most transcribed 12,822 genes; this ranking fell to #739 (58%) of the highest average FPKM for cells grown on TCPS. *HTRA1* is a serine protease that plays a crucial role in neuronal maturation, possibly through downregulation of the TGF-*β* signalling pathway (Launay et al., 2008). *HTRA1* met the differential expression criteria with a log_2_FC = 2.2.

### Identification of CNS biomarkers in monolayer- and matrix-cultured NHA transcriptomes

**Figure 8:**
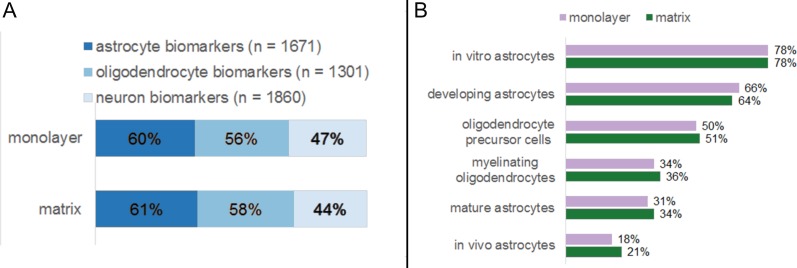
Percentages of established neural biomarkers that met RNA sequencing threshold criteria for NHA grown in monolayer and matrix environments. RNA sequencing data for NHA was compared to genes that were found to be enriched in neural cell types (A) and subtypes (B) by Cahoy et al. (2008).

We compared the transcriptome of NHA in monolayer and matrix conditions with established profiles for distinct CNS cell types (Fig. 8). As per Cahoy et al. (2008), a suite of marker genes specify several classes of murine neural cells, including astrocytes, oligodendrocytes, and neurons, (Cahoy et al., 2008). Of the genes that Cahoy et al. (2008) determined are significantly upregulated in astrocytes, 60 % and 61 % met our detectable threshold criteria in monolayer and matrix conditions, respectively (Fig. 8A) (Cahoy et al., 2008). Similarly, 56% and 58% of the genes that are designated as significantly upregulated in oligodendrocytes by Cahoy et al. (2008) met our detection criteria in in monolayer and matrix conditions, respectively (Fig. 8A) (Cahoy et al., 2008). For the genes found upregulated in neurons by Cahoy et al. (2008), 47% and 44% were detectable in monolayer and matrix-cultured NHA cells, respectively (Fig. 8A) (Cahoy et al., 2008).

We further probed for cellular heterogeneity in NHA cultures using marker genes for distinct CNS cell subtypes. Cahoy et al. (2008) developed subset profiles (80 genes each) of the most upregulated genes in specific classes of astrocytes (*in vitro*, *in vivo*, developing, and mature) and oligodendrocytes (precursors, myelinating) (Cahoy et al., 2008). When we undertook a more detailed analysis of the NHA transcriptome in monolayer and matrix conditions using these gene lists, we observed that markers for all CNS cell subtypes were present in similar amounts for both monolayer- and matrix- cultured NHA. However, the relative amount of NHA expressed genes varied between subtypes. For example the subtype *in vitro* astrocytes comprised the highest number of biomarker genes (monolayer, 78%; matrix, 78%; Fig. 8B), as compared to myelinating oligodendrocytes, where a fewer number of biomarkers were detectable (monolayer, 34%; matrix, 36%; Fig. 8B).

### Ontological analysis of differentially expressed genes with DAVID

**Figure 9:**
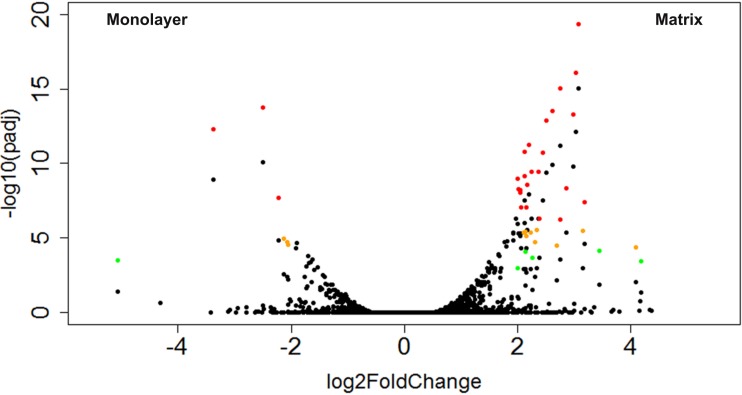
Volcano plot of CTSs that fulfilled expression threshold criteria. The log2 transformed fold change between monolayer and matrix environments is plotted against the negative log10 of the *p* value. The CTSs that were found to be significantly differentially expressed using our criteria of log2 fold change ≥ 2 and adjusted *p* values ≤ 0.1, 0.01, or 0.001 are shown in green, yellow, or red, respectively.

A total of 43 CTSs met our criteria for differentially expressed genes (DEGs; Table 2). Of these DEGs, 16% were upregulated in monolayer-cultured NHA, and 84% were upregulated in cells cultured with PuraMatrix™ (Fig. 9). DAVID analysis did not group the DEGs from TCPS-cultured cells into any clusters (Huang, Sherman & Lempicki, 2009a; Huang, Sherman & Lempicki, 2009b). In contrast, DAVID analysis assigned the DEGs upregulated in hydrogel-cultured cells into 9 significant clusters (enrichment score > 1.3) which we ranked 1–9 with the highest enrichment score corresponding to cluster 1 (Table 3 and File S7) (Huang, Sherman & Lempicki, 2009a; Huang, Sherman & Lempicki, 2009b).

**Table 2:** Differentially Expressed Genes. CTSs with log_2_ transformed fold change values ≥|2|.

DEG	log_2_ FC	*p* adj
**Upregulated in matrix**		
CHI3L1^*^	4.18	4.40E−02
TNFAIP8L3^**^	4.10	8.99E−03
SPP1^*^	3.44	1.31E−02
AREG^***^	3.19	2.56E−05
A2M^**^	3.16	1.07E−03
GPNMB^***^	3.08	8.84E−16
HEY2^***^	3.03	8.04E−13
PTGS2^***^	2.98	1.72E−10
CDH23^***^	2.86	4.33E−06
BMF^***^	2.76	6.30E−12
SLC16A6^***^	2.75	2.65E−04
ANGPTL4^**^	2.70	7.23E−03
SPOCK3^***^	2.62	1.18E−10
CYGB^***^	2.52	3.98E−10
PIEZO2^***^	2.45	3.06E−08
EREG^***^	2.39	2.27E−04
PTGDS^***^	2.36	5.16E−07
NPTX1^**^	2.30	4.15E−03
SLITRK5^*^	2.26	2.97E−02
EPHB1^***^	2.25	5.16E−07
HMOX1^**^	2.24	1.27E−03
UNC5B^***^	2.20	1.12E−08
CLDN14^***^	2.18	2.86E−06
NR4A2^***^	2.16	4.89E−05
IL1A^**^	2.15	2.08E−03
RDH10^*^	2.14	1.51E−02
CYP1B1^**^	2.14	1.20E−03
HTRA1^***^	2.13	3.06E−08
NRG3^***^	2.13	9.55E−07
RAB27B^**^	2.11	1.26E−03
ITGA8^***^	2.06	4.89E−05
IL21R^***^	2.04	7.21E−06
ST8SIA4^***^	2.04	5.27E−06
GPR68^***^	2.02	5.22E−06
COL21A1^*^	2.00	9.67E−02
DUSP4^***^	2.00	1.22E−06
**Upregulated in monolayer**		
NPPB^*^	−5.07	4.09E−02
ACTG2^***^	−3.38	1.29E−09
MAMDC2^***^	−2.50	8.73E−11
DES^***^	−2.22	1.48E−05
GCNT4^**^	−2.13	2.70E−03
DYSF^**^	−2.06	4.28E−03
EGF^**^	−2.05	6.38E−03

**Notes.**

Significance levels marked by Benjamini-Hochberg adjusted *p* values.

* <0.1.

** <0.01.

*** <0.001.

### Predicted protein interactions for DEGs

**Figure 10:**
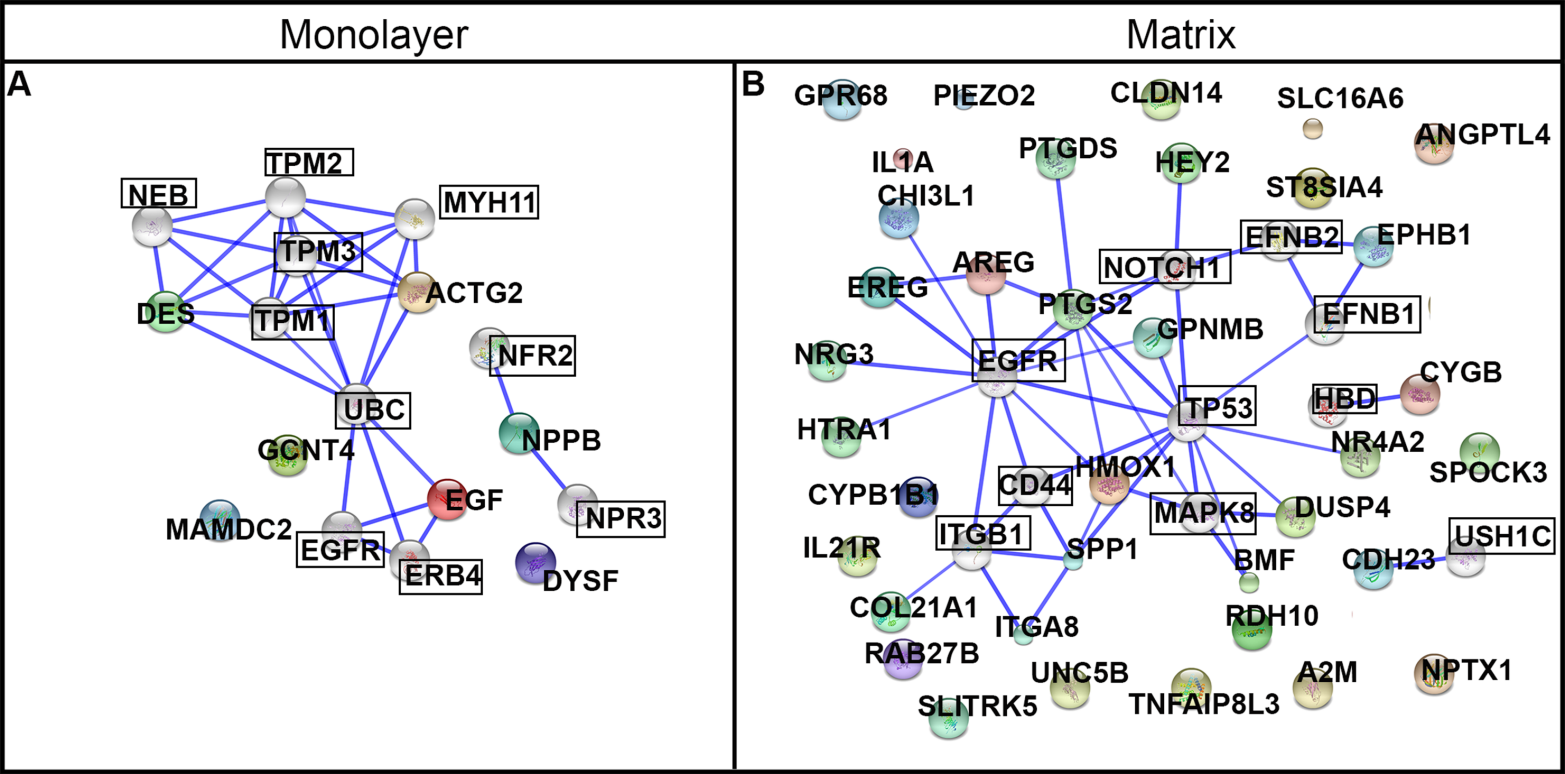
Protein networks predicted by STRING analysis. DEGs were used as input for the STRING database using the high stringency setting. Ten interacting nodes (boxed) are shown for networks derived from DEGs upregulated in monolayer (A) and matrix (B) conditions (not boxed).

We used the STRING database to conduct a deeper exploration of the network of protein interactions for the DEGs. We configured STRING for ten additional interacting nodes beyond the 7 and 36 DEGs used as input for the monolayer-cultured and matrix-cultured cells, respectively (Table 2 and Fig. 10). For cells cultured on TCPS, the top 10 interacting proteins predicted by STRING were EGFR, NPR3, UBC, ERBB4, NEB, MYH11, TPM2, NPR2, TPM1, and TPM3 (Fig. 10A). The proteins encoded by the genes upregulated in cells cultured with PuraMatrix™ were predicted to interact with ITGB1, EFNB1, NOTCH1, MAPK8, EFNB2, USH1C, HBD, CD44, EGFR, and TP53 (Fig. 10B).

The predicted functional significance of the protein interactors was explored with GeneCards (Safran et al., 2010). The CNS emerged as a common theme, and was set as a goal for further exploration. Seven of the top ten interacting proteins for matrix-upregulated genes had an established role in the nervous system that was mentioned in GeneCards (ITGB1, migration and survival of primary oligodendrocytes; EFNB1, mitigates commissural axons and growth cones; NOTCH, differentiation of Bergmann glia, represses neuronal differentiation; TP53, implicated in NOTCH signaling cascade, response to oxidative stress; MAPK8, controls neurite elongation in cortical neurons; EFNB2, constrains longitudinally projecting axons, angiogenesis, neuronal development; USH1C, part of the network that mediates mechanotransduction in cochlear hair cells). In contrast, exploring data on GeneCards uncovered an established role in the nervous system for only one of the top ten interacting proteins for monolayer-upregulated genes (ERBB4, nervous system development) (Safran et al., 2010).

### Significance of differentially expressed genes for CNS function

**Figure 11:**
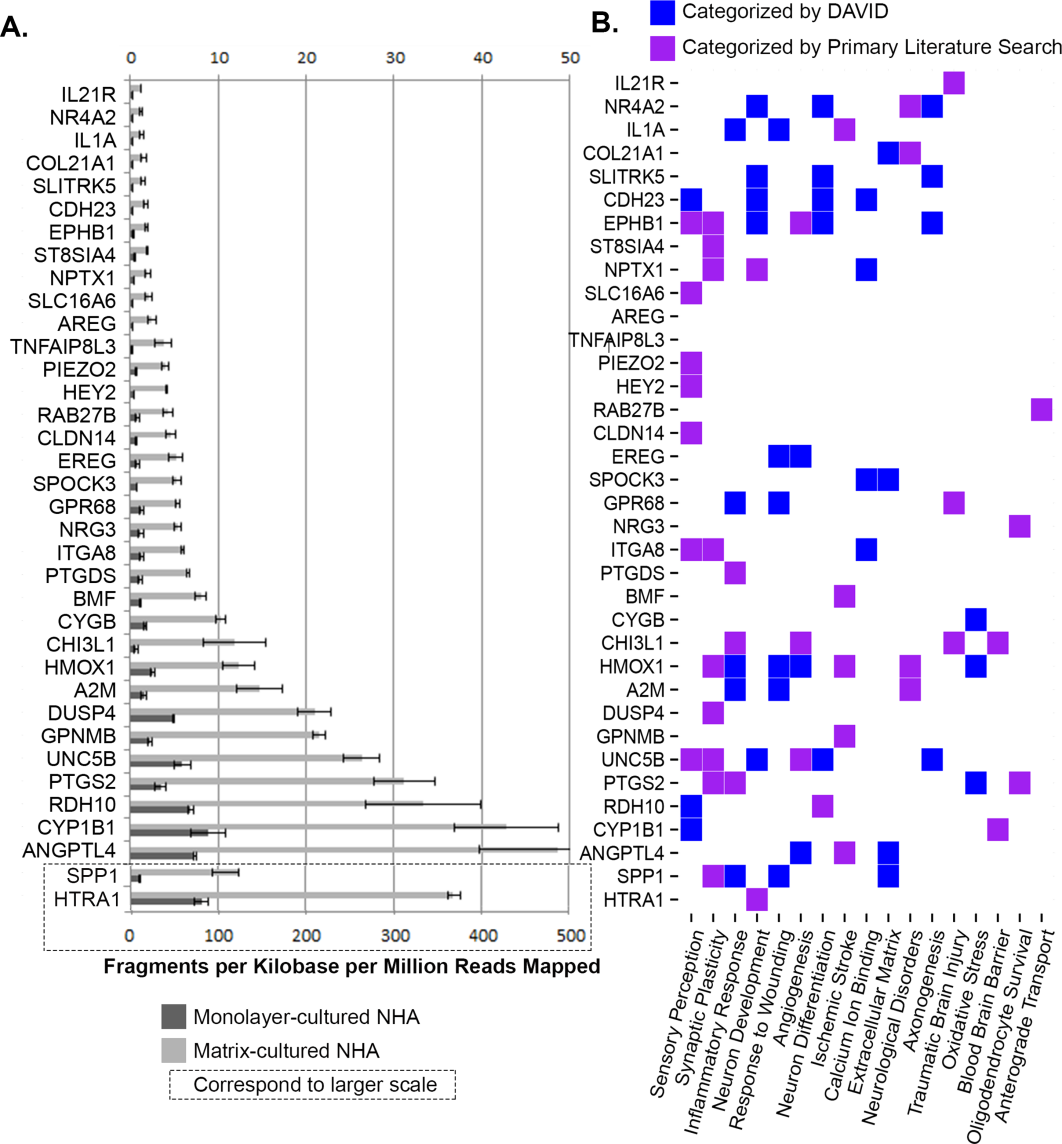
Expression levels and ontological characterization of genes upregulated in hydrogel-cultured NHA. Normalized expression levels (FPKM) for hydrogel-upregulated genes are shown for monolayer (dark grey) and matrix (light grey) cultures (A). Functional classification categories assigned to hydrogel-upregulated genes by DAVID (blue) or through manual curation (purple). Categories are displayed left to right in order from those with the greatest number of genes (synaptic plasticity; *n* = 10) to the least number of genes (anterograde transport, *n* = 1).

The relevance of the DEGs in the nervous system was probed through pooled information sources (DAVID, GeneCards) as well as primary literature queries (PubMed) of individual genes along with the terms “*brain*” and “*neural*.” One of the seven genes upregulated in monolayer-cultured cells had a previously established neural function. Of the 36 genes upregulated in hydrogel-cultured cells, 34 were assigned to CNS function either through manual curation or functional clustering by DAVID (Fig. 11) (Huang, Sherman & Lempicki, 2009a; Huang, Sherman & Lempicki, 2009b; Safran et al., 2010). Of the hydrogel-upregulated genes, ∼42% (*n* = 15) have an established role in response to neural insult and correspond to processes such as oxidative stress (and/or angiogenesis), ischemic stroke, and traumatic brain injury. Approximately 39% (*n* = 14) of the genes upregulated in matrix-cultured cells are involved in neuronal differentiation and growth, synapse formation, and neural plasticity.

**Table 3:** Significant Clusters of Differentially Expressed Genes. DAVID Functional analysis of differentially expressed genes (log2 fold change ≥ 2 and adjusted *p* values ≤ 0.1) resulted in nine significant functional clusters (enrichment score > 1.3).

Cluster	Score	Description	Genes
1	5.6	Glycoprotein, signal, signal peptide, glycosylation site: N-linked	A2M, NRG3, PTGS2, SPOCK3, EREG, PTGDS, ITGA8, ST8SIA4, AREG, GPNMB, SLITRK5, IL1A, COL21A1, IL21R, CHI3L1, GPR68, EPHB1, NPTX1, UNC5B, ANGPTL4, CDH23, SPP1
2	3.5	Extracellular region, extracellular space, secreted	A2M, SPOCK3, EREG, COL21A1, HTRA1, HMOX1, CHI3L1, AREG, IL1A, SPP1, ANGPTL4
3	2.0	ErbB signalling pathway, domain: EGF-like, EGF, EGF-like region: conserved site, EGF-like, EGF-like: type 3, growth factor	NRG3, PTGS2, EREG, AREG
4	1.9	Cell morphogenesis involved in neuron differentiation, cell projection organization, neuron projection morphogenesis, neuron development, axonogenesis	UNC5B, ITGA8, NR4A2, SLITRK5, EPHB1, CDH23
5	1.6	Response to (wounding, inflammation, extracellular signal, nutrient levels, steroid hormone stimulus, endogenous stimulus, organic substance, inorganic substance), organelle lumen, membrane-enclosed lumen	GPR68, IL1A, DUSP4, A2M, CYP1B1, PTGS2, HMOX1, NR4A2, SPP1
6	1.6	Blood vessel morphogenesis, vasculature development, regulation of cell proliferation, angiogenesis	PTGS2, EREG, HMOX1, HEY2, IL1A, SPP1, ANGPTL4
7	1.6	Extracellular matrix, proteinaceous extracellular matrix	SPOCK3, COL21A1, SPP1, ANGPTL4
8	1.5	Ossification, biomineral formation, bone development, skeletal system development	PTGS2, GPNMB, SPP1
9	1.4	Positive regulation’ of (multicellular organismal process, cytokine biosynthetic process, cell communication, macromolecule biosynthetic process, cellular biosynthetic process, macromolecule metabolic process, regulation of cytokine production, cell proliferation	PTGS2, EREG, HMOX1, HEY2, IL1A, SPP1

**Figure 12:**
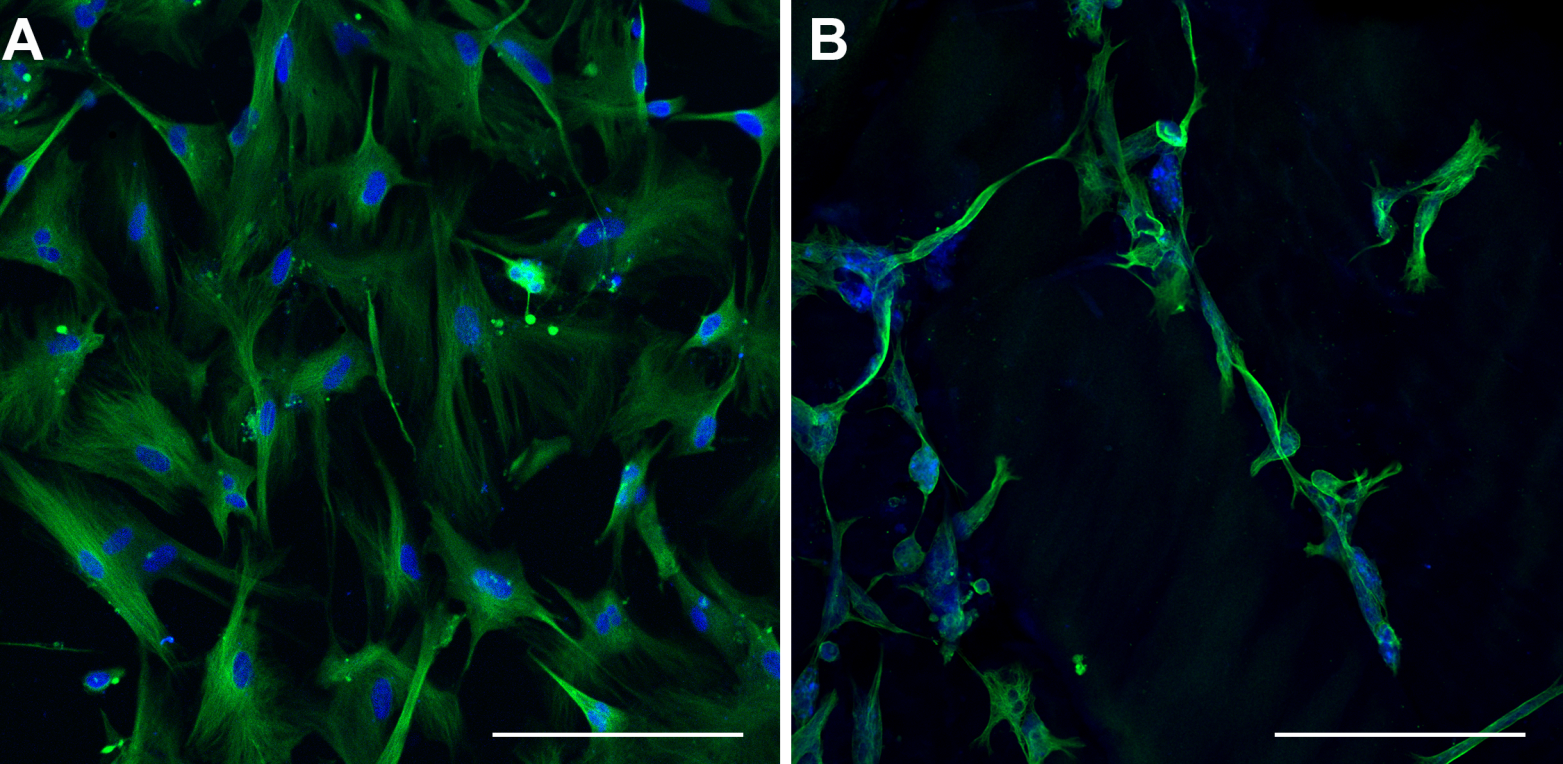
Class III *β*-tubulin Immunocytochemistry. Optical sections of NHA samples cultured in monolayer (A) and matrix (B) environments for 5 days. Cells were fixed prior to staining class III *β*-tubulin (green) and nuclei (blue) with anti –TUBB3 antibody and Hoescht 33342, respectively. Scale bar = 200 µm.

Moreover, over a quarter (*n* = 10) of the genes upregulated in matrix-cultured NHA play a role in sensory perception. The manually curated associations are described in more detail below.

#### Hydrogel-upregulated genes involved in stress responses

Manual curation and the bioinformatics resource DAVID suggest that fifteen genes upregulated in hydrogel-cultured cells participate in the CNS responses to inflammation, oxidative stress, traumatic brain injury, and ischemic stroke (Fig. 11). The collective information resources DAVID and GeneCards linked seven of these genes to the inflammatory response of the CNS, including *PTGS2, HMOX1, A2M, SPP1, GPR68, and IL1A* (Huang, Sherman & Lempicki, 2009a; Huang, Sherman & Lempicki, 2009b; Safran et al., 2010). Other experiments have provided evidence that *CHI3L1, GPR68* and *IL1A* are involved in the response to traumatic brain injury (Hua et al., 2011; Schneider et al., 2012; Wiley et al., 2015). DAVID associated ‘*response to oxidative stress*’ with *HMOX1, CYGB,* and *PTGS2* (File S7) (Huang, Sherman & Lempicki, 2009a; Huang, Sherman & Lempicki, 2009b). DAVID assigned the GO term ‘*angiogenesis*’ to *HMOX1, EREG*, and *ANGPTL4* (File S7) (Huang, Sherman & Lempicki, 2009a; Huang, Sherman & Lempicki, 2009b). Analysis of function in GeneCards revealed that *EPHB1* and *UNC5B* also regulate angiogenesis (Safran et al., 2010). Literature reports that *ANGPTL4* upregulation corresponds to increased vascularization after stroke (Schipper et al., 2014). Results from prior studies show that *IL21R, BMF*, and *GPNMB* correspond to mediators of injury following ischemic stroke (Clarkson et al., 2014; Nakano et al., 2014; Pfeiffer et al., 2014).

#### Hydrogel-upregulated genes involved in neuronal growth and differentiation

Reports of previous findings and DAVID functional analysis indicate that fourteen of the genes upregulated in hydrogel-grown NHA are associated with neuronal differentiation and growth, synapse formation, and neural plasticity (Fig. 11). The primary literature and content of GeneCards suggest that *ST8SIA4, PTGS2*, and *HMOX1* are involved in neural plasticity, along with *DUSP4*, indirectly, through interactions with *GRIN1* and *GRIN2A* (Hascalovici et al., 2009; Safran et al., 2010; Chen et al., 2013). Previous studies provide evidence that *RDH10* is involved in neuronal differentiation during embryonic development, and *UNC5B*, a netrin receptor, mediates the repulsion of growth cones in the axon during nervous system development (Kaur et al., 2007; Chatzi, Cunningham & Duester, 2013). Experiments have identified *NPTX1* as a marker of commitment to neural lineage that is involved in synaptic remodelling (Boles et al., 2014).

Analysis of GeneCards revealed that *NR4A2* is a transcriptional regulator that plays a role in the differentiation of meso-diencephalic dopaminergic neurons (Safran et al., 2010). *HTRA1* is noted in the literature for its involvement in neuronal maturation, possibly through downregulation of TGF-*β* (Launay et al., 2008). GeneCards analysis revealed that *SLITRK5* and *ITGA8* modulate neurite growth (Safran et al., 2010). Researchers have indicated that *SPP1* interacts with *ITGA8*, and its expression pattern corresponds to the development of finger dexterity (Yamamoto et al., 2013). Review of GeneCards uncovered that *EPHB1* plays an important role in synapse formation and the maturation of dendritic spines (Safran et al., 2010).

#### Hydrogel-upregulated genes involved in sensory processes

Ontological characterization with DAVID and review of the primary literature led us to conclude that ten of the genes upregulated in hydrogel-cultured cells play a role in sensory processes such as vision, audition, and touch sensation (Fig. 11). DAVID assigned the GO term ‘*sensory perception*’ to three genes: *CDH23, CYP1B1,* and *RDH10* (File S7) (Huang, Sherman & Lempicki, 2009a; Huang, Sherman & Lempicki, 2009b). A deeper analysis with GeneCards illustrated that *CDH23* is part of the complex that mediates mechanotransduction in cochlear hair cells, *CYP1B1* is predicted to be involved in eye development and differentiation, and *RDH10* mutants can have visual or auditory phenotypes (Safran et al., 2010). GeneCards also elucidated that *EPHB1* regulates retinal axon guidance, and *ITGA8* regulates neurite growth in sensory neurons (Safran et al., 2010). Results of previous studies provide evidence that *HEY2* regulates the differentiation of mammalian auditory hair cells, whereas *SLC16A6* is expressed in cochlear sensory hair cells and plays a role in deafness (Benito-Gonzalez & Doetzlhofer, 2014; Girotto et al., 2014). Analysis with GeneCards illustrated that *CLDN14* is also associated with sensorineural deafness, and *PIEZO2* is a component of the mechanotransduction apparatus that senses light touch stimuli (Safran et al., 2010). Studies have shown that *UNC5B* is expressed primarily in sensory structures such as the eye, ear, and brain (Kaur et al., 2007).

#### Other CNS functions of hydrogel-upregulated genes

Manual curation of gene function through review of the primary literature and the information on GeneCards revealed additional CNS functions for genes upregulated in matrix-cultured NHA, including involvement with the blood brain barrier (BBB) and astrocyte-specific genes, as well as genes associated with neurological disorders and other functions. In particular, *CLDN14* is an essential component the tight junctions comprising the BBB. The reported high expression levels for *CYP1B1* and *CH31L1* in the BBB suggest a role for these genes in BBB function (Safran et al., 2010; Decleves et al., 2011; Bjørnbak et al., 2014). *CHI3L1* is also an astroglial lineage marker, and *AREG* acts as a growth factor and mitogen in astrocytes (Safran et al., 2010; Bjørnbak et al., 2014). *RAB27B* is involved in axonal anterograde transport (Arimura et al., 2009). *SPOCK3* is a calcium-binding extracellular proteoglycan that is specifically expressed in the brain (Hartmann et al., 2013). *PTGS* plays a role in sedation, NREM sleep, pain, and may play an anti-apoptotic role in oligodendrocytes (Safran et al., 2010). *NRG3* is also thought to be a survival factor for oligodendrocytes (Safran et al., 2010). In addition, four genes associated with neurological disorders were significantly upregulated in hydrogel-cultured cells (NR4A2, Parkinson’s disease; A2M & HMOX1, Alzheimer’s; COL21A1, atypical psychosis) (Safran et al., 2010; Kanazawa et al., 2013; Schipper & Song, 2015).

#### CNS function of TCPS-upregulated genes

For NHA cultured on TCPS, querying the neural relevance of the DEGs in the nervous system through pooled information sources (DAVID, GeneCards) as well as primary literature queries (PubMed) of individual genes along with the terms “brain” and “neural” resulted in a smaller proportion of genes with relevance to the CNS. Of the 7 genes upregulated in TCPS-cultured cells, one gene (EGF) was found to have a previously established neural function. Analysis of GeneCards revealed that EGF can induce neurite outgrowth (Safran et al., 2010). One in seven corresponds to approximately 14% of the genes upregulated in TCPS-cultured cells with a previously established function in the CNS. In contrast, 94% of hydrogel-upregulated genes were known to play a role in the CNS.

### Immunocytochemical detection of class III *β*-tubulin

In order to characterize the morphological differences observed in TCPS- and hydrogel- cultured NHA, class III *β*-tubulin was labelled using immunocytochemical methods. A pervasive, positive stain for class III *β*-tubulin was observed throughout the cell body of every cell cultured on TCPS (Fig. 12A). This result is consistent with the upregulated expression of class III *β*-tubulin in human fetal brain tissue (Safran et al., 2010). In contrast, a more heterogeneous staining pattern for class III *β*-tubulin was seen in matrix-cultured NHA (Fig. 12B). Some cells appeared unlabelled, and there was a spatially variable pattern of fluorescence in the cells that stained positively. In particular, long protrusions from the cell body of some cells appeared to label more intensely than other regions of the cell. Three different individuals blindly grouped optical sections and maximum confocal projections into two conditions and verified the labelling patterns seen in the two groups.

## Discussion

After five days in culture, the morphology of hydrogel-cultured NHA resembled what is typically observed in primary mouse neuron-astrocyte co-cultures, while monolayer-cultured cells retained the phase dark appearance that typifies astrocyte cultures (Serrano & Schimke, 1990; Abd-el-basset, 2013). The change in cellular organization was accompanied by a difference in the staining pattern of class III *β*-tubulin between the two conditions. In monolayer-cultured NHA, the tubulin stain was prevalent throughout all cells, whereas in matrix-cultured NHA, heterogeneous labelling was observed. These findings are consistent with the results of experiments by Azizi & Krynska (2013), who developed a protocol to induce neuronal differentiation in NHA by altering media, growth factors, and using a hydrogel comprising natural extracellular matrix proteins (Azizi & Krynska, 2013). In contrast, we found that culturing cells with the synthetic hydrogel PuraMatrix™, and no change in media or growth factors, was sufficient for the induction of morphological and transcriptomic differences. The limited bioreactivity of PuraMatrix™ suggests that changes in our NHA cultures may be attributed, at least in part, to the mechanical properties or structural organization of the growth environment (Holmes et al., 2000; Zhang, Ellis-behnke & Zhao, 2005; Sur et al., 2012). The altered morphology we observed in NHA cultured with PuraMatrix™ is congruent with previous findings where neuronal differentiation of immature cell types has been shown to depend primarily upon stiffness of the culture substrate (Teixeira et al., 2009; Sur et al., 2012).

Next generation RNA sequencing provides a comprehensive view of expressed transcripts and transcripts that are differentially regulated between culture conditions. Therefore, for insight into the key molecular players that direct the cellular response to altered substrate elasticity, we undertook next generation RNA sequencing experiments of TCPS- and hydrogel-cultured NHA. The adherence to stringent criteria for RNA integrity (RIN ≥ 8.8) facilitated the collection of sequence data with Phred scores >20. Transcriptomic analysis revealed 43 genes that were significantly differentially expressed between monolayer and matrix conditions.

Characterization of the ontological terms associated with the most expressed transcripts in both conditions revealed, as expected, an assortment of terms associated with basic cellular function. When the DEGs in both conditions were evaluated with STRING and GeneCards, hydrogel-upregulated genes were predicted to interact with proteins that have a higher percentage of nervous system relevance (70%) as compared to the proteins predicted to interact with TCPS-upregulated genes (10%). This trend was also observed in the ontological characterization of the DEGs. Manual curation and ontological characterization of DEG function with DAVID and GeneCards revealed that 94% of the genes upregulated in matrix-cultured NHA had an established CNS role, compared to 14% of genes upregulated in monolayer-cultured NHA. Functional analysis of the hydrogel-upregulated genes led to our classification scheme of three “umbrella” categories: (1) response to neural insult (15 genes), neuronal differentiation and/or neural plasticity (14 genes), and sensory perception (10 genes).

We propose that the genes involved in neuronal differentiation and maturation contribute to the molecular basis of the altered morphology and structural reorganization observed in hydrogel-cultured NHA. In particular, *UNC5B*, *HTRA1*, *ITGA8*, and *SPP1* would make intriguing candidates for future studies due to their relatively high expression levels and fold changes as compared with other genes in this umbrella category. Moreover, two of the top five most upregulated genes in matrix-cultured NHA, *CHI3L1* and *AREG*, have an established role in astrocyte growth and differentiation. The expression of markers for neuronal and glial maturity in hydrogel-cultured NHA suggests that differentiation of NHA into mature neurons and astrocytes is enhanced when cultured in a hydrogel environment. Results are consistent with previous reports of neuronal and astrocytic enhancement on soft substrates (Teixeira et al., 2009).

The differences between TCPS and hydrogel- cultured NHA warrant further investigation into the structural and molecular bases of our findings. For example, the observation that a number of hydrogel-upregulated genes are involved in sensory perception (*n* = 10) inspires functional studies with electrophysiological techniques or calcium imaging. Electrophysiological approaches could also be used to evaluate changes in synaptic plasticity, another functional category comprising several (*n* = 9) hydrogel-upregulated genes. In addition, the number of hydrogel-upregulated genes involved in the inflammatory response (*n* = 8) prompts experiments that can measure secreted inflammatory and anti-inflammatory mediators. Extended live imaging experiments can be used to determine whether the structural reorganization of NHA is due to migration or local division, while transmission electron microscopy can be applied to uncover ultrastructural features of cellular and subcellular organization within the hydrogel environment. Moreover, characterization of the structure of the hydrogel with scanning electron microscopy, as well as measuring the elasticity of the hydrogel through atomic force microscopy, could contribute to the design of hydrogels for therapeutic applications.

## Conclusion

NHA cultured with the peptide hydrogel PuraMatrix™ undergo a morphological and transcriptomic shift in the absence of additional growth factors. Next-generation RNA sequencing and ontological analyses revealed key molecular players involved in maturation and differentiation of neurons and glia that were upregulated in hydrogel-cultured NHA. The upregulation of gene biomarkers which signify neuronal and glial maturity in hydrogel-cultured NHA suggests that differentiation of NHA into mature neurons and astrocytes can be stimulated when cells are cultured in a hydrogel environment.

##  Supplemental Information

10.7717/peerj.2829/supp-1 File S1 Coding transcript sequences with FPKM values of NA Normalized FPKM values for monolayer and matrix samples. Coding transcript sequences removed from downstream analyses due to values of NA (not applicable).

10.7717/peerj.2829/supp-2 File S2 Coding transcript sequences with FPKM values of 0 Normalized FPKM values for monolayer and matrix samples. Coding transcript sequences removed from downstream analyses due to sub-threshold values of 0

10.7717/peerj.2829/supp-3 File S3 Coding transcript sequences with sub-threshold values Normalized FPKM values for monolayer and matrix samples. CTSs removed from downstream analyses due to sub-threshold values of 0.1–1.

10.7717/peerj.2829/supp-4 File S4 Noncoding RNA sequences Normalized FPKM values for monolayer and matrix samples. Noncoding RNA sequences removed from downstream analyses

10.7717/peerj.2829/supp-5 File S5 Detectable coding transcript sequences Normalized FPKM values for monolayer and matrix samples: All coding transcript sequences used in downstream analyses

10.7717/peerj.2829/supp-6 File S6 DAVID clustering of common highly expressed sequences Normalized FPKM values for monolayer and matrix samples. DAVID clustering results of highest 10% of FPKM values for coding transcript sequences common to monolayer and matrix samples.

10.7717/peerj.2829/supp-7 File S7 DAVID clustering of matrix-upregulated genes DAVID clustering results of matrix-upregulated genes designated by DeSeq using differential expression criteria of FC >2 and *p* adjusted <0.1

10.7717/peerj.2829/supp-8 Figure S1 RNA quality evaluation Electropherograms of RNA samples used for Illumina library preparation

10.7717/peerj.2829/supp-9 Figure S2 RNA-seq quality evaluation by Phred score Average Phred score per nucleotide position scored using FastQC

10.7717/peerj.2829/supp-10 Figure S3 RNA-seq quality evaluation by nucleotide composition Total percentage of nucleotide composition at each read position scored using FastQC

## List of abbreviations

%: percent
^∘^C: degrees Celsius
µl: microliter
3D: tridimensional
bp: base pair
CCD: charge-coupled device
cm^2^: square centimetre
CNS: central nervous system
CO_2_: carbon dioxide
CTS: coding transcript sequence
DAVID: Database for Annotation, Visualization, and Integrated Discovery
DEG: differentially expressed gene
DNA: deoxyribonucleic acid
FPKM: fragments per kilobase of exon per million reads mapped
GO: Gene Ontology Consortium
G: gravity
mOsm: milliosmole
ng: nanograms
NHA: normal human astrocytes
RNA: ribonucleic acid
RNA-seq: RNA sequencing
RIN: RNA integrity number
PBS: phosphate buffered saline
PCR: polymerase chain reaction
PE: paired end
qPCR: quantitative polymerase chain reaction
BSA: bovine serum albumin
TCPS: tissue culture polystyrene
UCSC: University of California Santa Cruz

## Additional Information and Declarations

### DNA Deposition

The following information was supplied regarding the deposition of DNA sequences: GEO: GSE81995.

### Data Availability

The following information was supplied regarding data availability: Raw and processed data available at GEO Accession number GSE81995
Code on GitHub: https://github.com/bleuknight/Normal_Human_Astrocyte_3D.
