# Supplementary figures and images for: Hydrogel scaffolds promote neural gene expression and structural reorganization in human astrocyte cultures
Source: PeerJ. 2017 Jan 11;5:e2829. doi: 10.7717/peerj.2829 (PMC5234438; doi:10.7717/peerj.2829)

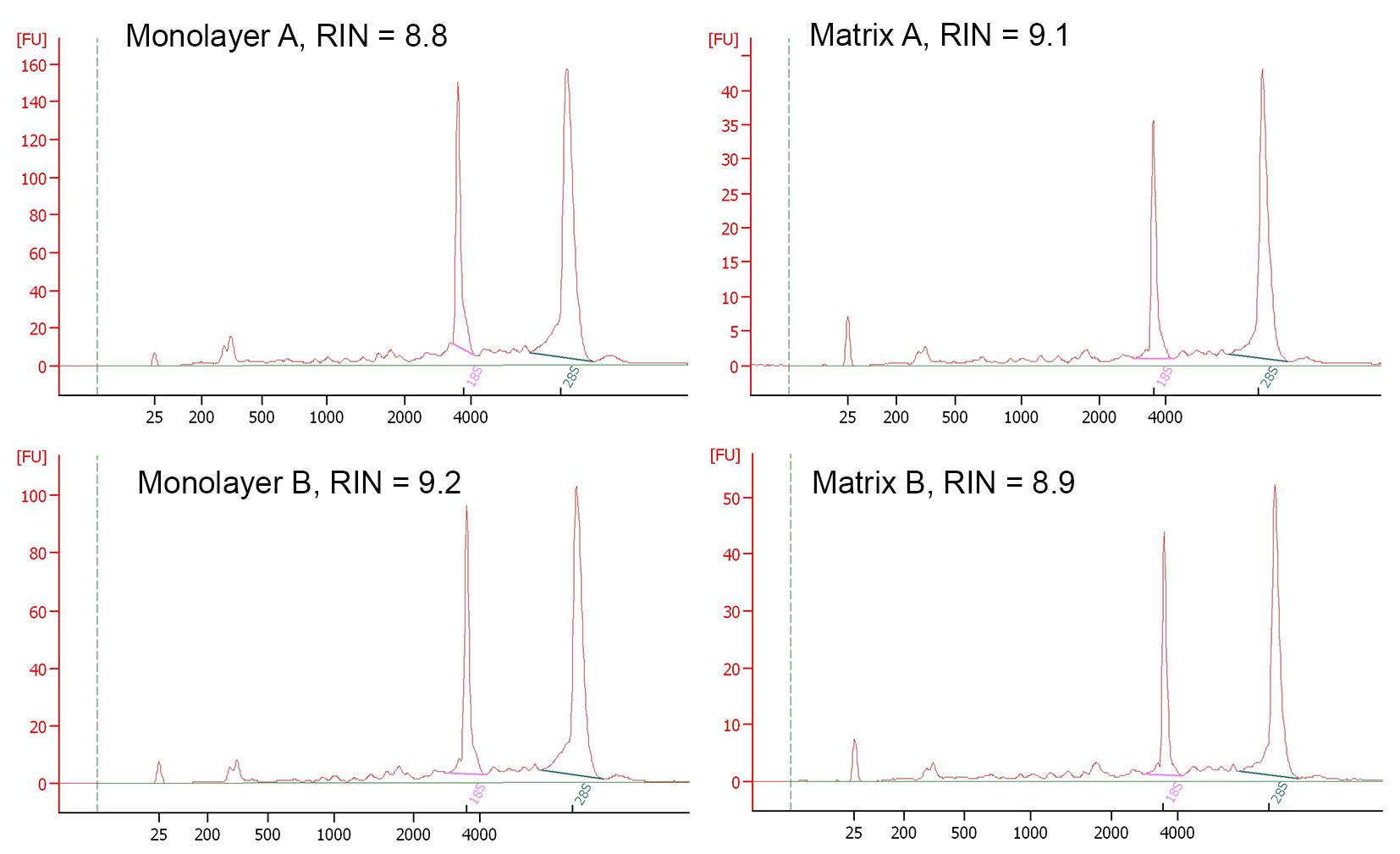

Supplement: Figure S1 — Electropherograms of RNA samples used for Illumina library preparation [file peerj-05-2829-s008.jpg]

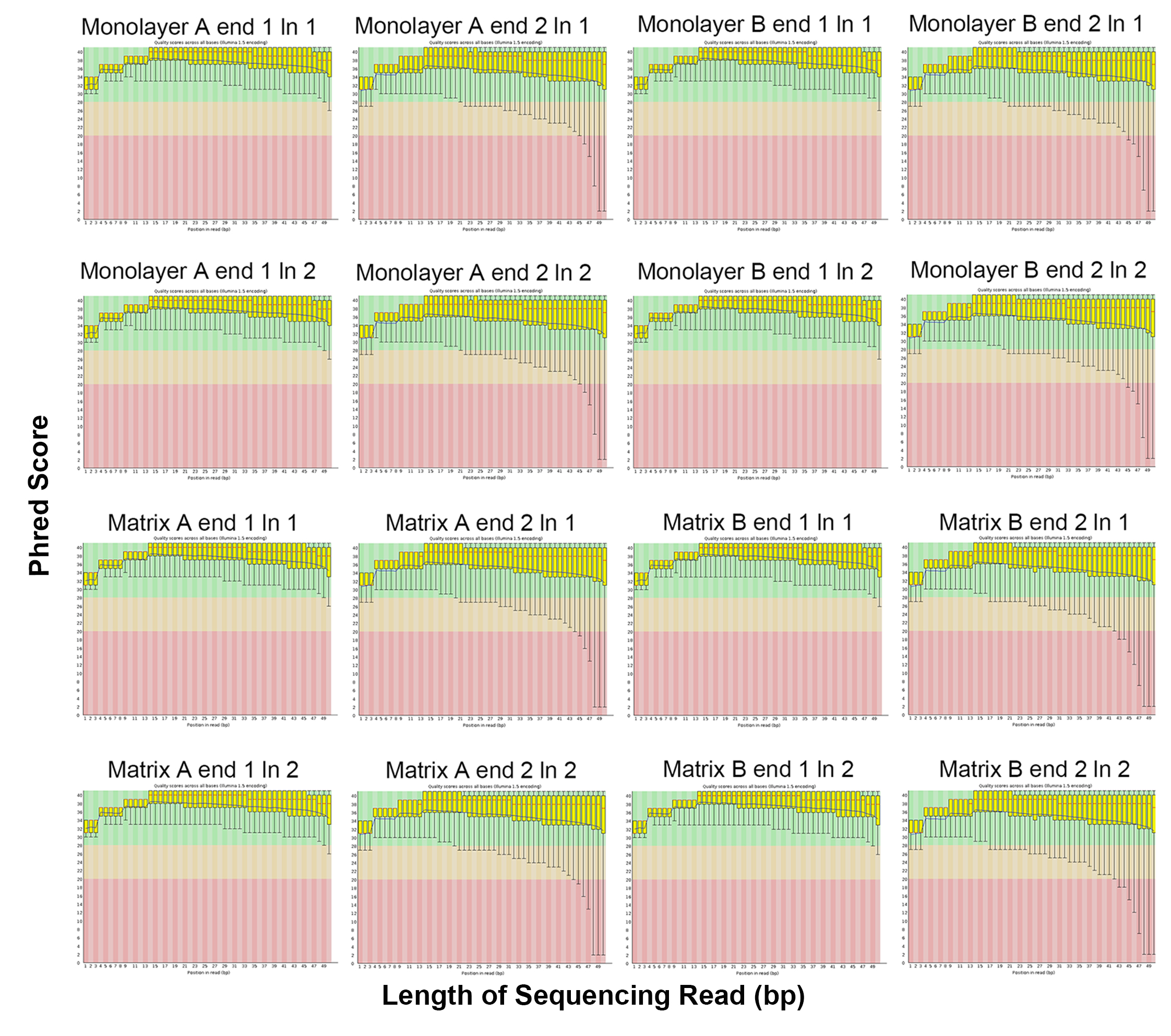

Supplement: Figure S2 — Average Phred score per nucleotide position scored using FastQC [file peerj-05-2829-s009.jpg]

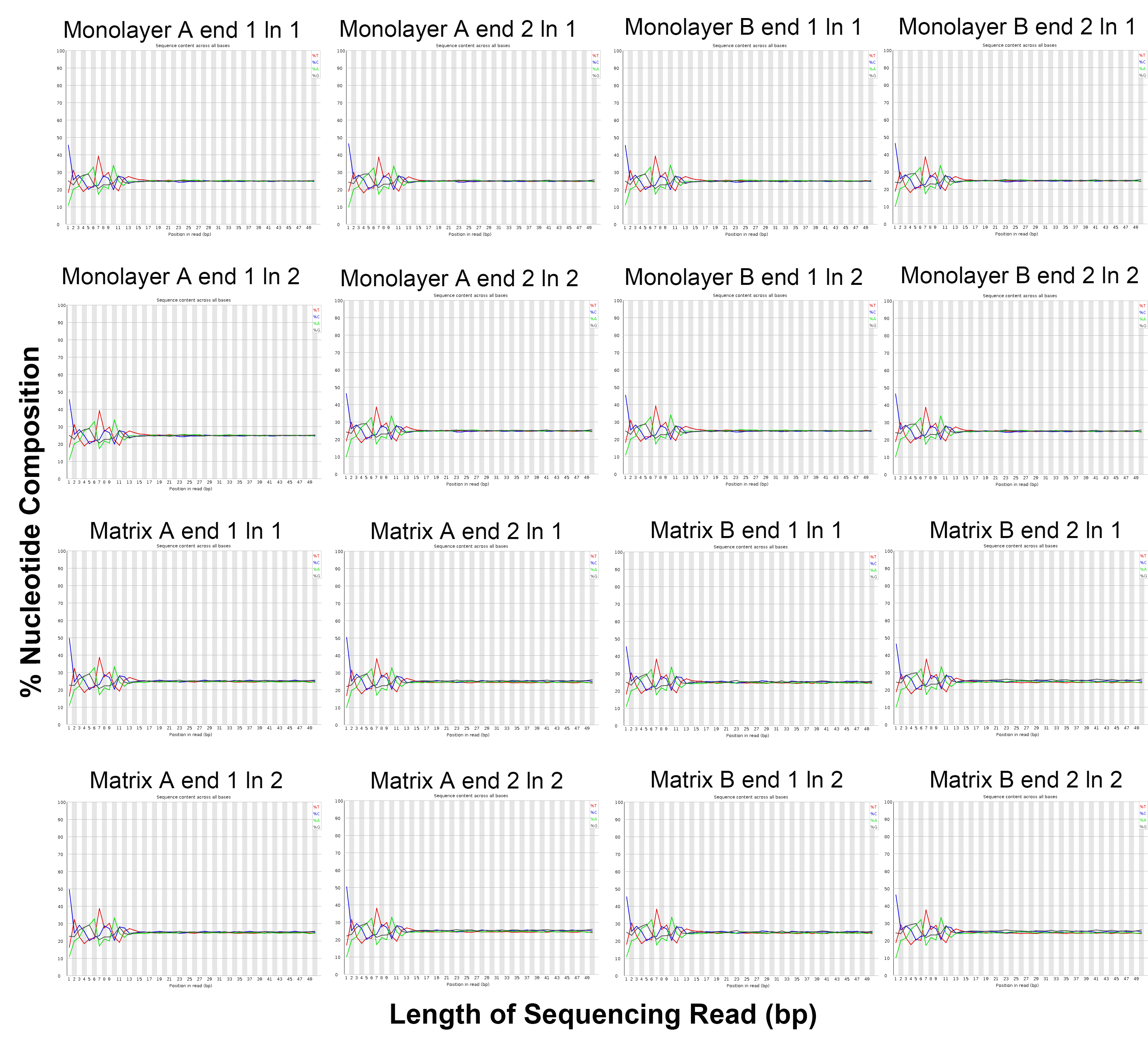

Supplement: Figure S3 — Total percentage of nucleotide composition at each read position scored using FastQC [file peerj-05-2829-s010.jpg]
